# Cultivating Prognostic Awareness in Acute Care: The Role of Social Work in Interprofessional Palliative Care Teams

**DOI:** 10.1093/geroni/igaf122.2165

**Published:** 2025-12-31

**Authors:** Arden O’Donnell, Juliet Jacobsen, Rennie Bimman, Judith Gonyea

**Affiliations:** Boston University, Boston, Massachusetts, United States; Massachusetts General Hospital, Boston, Massachusetts, United States; Massachusetts General Hospital, Boston, Massachusetts, United States; Boston University, Boston, Massachusetts, United States

## Abstract

For patients with life-limiting illnesses, informed medical decision-making depends on an understanding of disease prognosis and trajectory. Prognostic awareness—the ability to recognize and integrate knowledge about one’s illness—has been associated with reduced psychological distress, improved quality of life and enhanced bereavement outcomes. There is growing recognition that cultivating prognostic awareness is an iterative, interprofessional process involving multiple disciplines. Using interpretive description methodology and thematic analysis, this study explores the clinical skills PSWs use to support prognosis-related discussions. The Prognostic Awareness Integration Scale (PAIS) serves as the conceptual framework for understanding both the cognitive and emotional processing of prognosis. Semi-structured interviews conducted with 17 matched pairs of palliative social workers and physicians across the United States. Findings affirm the PSW’s role in cultivating prognostic awareness, identifying specific interventions that support both cognitive understanding and emotional processing of serious illness discussions. PSWs enhance cognitive understanding by assessing knowledge gaps, providing education on disease trajectory, translating medical terminology, repeating key medical details, and offering anticipatory guidance. They also address emotional aspects by assessing coping capacities, evaluating the psychosocial impact of symptoms, modulating the pace and intensity of discussions, facilitating emotionally challenging conversations, centering emotions over medical details, and promoting a trauma-informed approach. The PAIS tool offers critical insights into the specific skills and strategies PSWs use to navigate complex, emotionally charged conversations about prognosis. The results of this study can be used in further defining the advanced skills of specialty palliative social work as well as in education and training.

